# Evolution and Comprehensive Analysis of DNaseI Hypersensitive Sites in Regulatory Regions of Primate Brain-Related Genes

**DOI:** 10.3389/fgene.2019.00152

**Published:** 2019-03-07

**Authors:** Yueer Lu, Xiao Wang, Hang Yu, Jianlin Li, Zhiqiang Jiang, Bangwei Chen, Yueqi Lu, Wei Wang, Chongyin Han, Ying Ouyang, Lizhen Huang, Chunbo Chen, Weidong Tian, Fei Ling

**Affiliations:** ^1^School of Biology and Biological Engineering, South China University of Technology, Guangzhou, China; ^2^State Key Laboratory of Genetic Engineering, Department of Biostatistics and Computational Biology, Collaborative Innovation Center of Genetics and Development, School of Life Sciences, Fudan University, Shanghai, China; ^3^Department of Critical Care Medicine, Guangdong General Hospital, Guangdong Academy of Medical Sciences, Guangzhou, China

**Keywords:** brain, regulatory region, DNaseI hypersensitive sites, evolution, primates

## Abstract

How the human brain differs from those of non-human primates is largely unknown and the complex drivers underlying such differences at the genomic level remain unclear. In this study, we selected 243 brain-related genes, based on Gene Ontology, and identified 184,113 DNaseI hypersensitive sites (DHSs) within their regulatory regions. To performed comprehensive evolutionary analyses, we set strict filtering criteria for alignment quality and filtered 39,132 DHSs for inclusion in the investigation and found that 2,397 (~6%) exhibited evidence of accelerated evolution (aceDHSs), which was a much higher proportion that DHSs genome-wide. Target genes predicted to be regulated by brain-aceDHSs were functionally enriched for brain development and exhibited differential expression between human and chimpanzee. Alignments indicated 61 potential human-specific transcription factor binding sites in brain-aceDHSs, including for CTCF, FOXH1, and FOXQ1. Furthermore, based on GWAS, Hi-C, and eQTL data, 16 GWAS SNPs, and 82 eQTL SNPs were in brain-aceDHSs that regulate genes related to brain development or disease. Among these brain-aceDHSs, we confirmed that one enhanced the expression of GPR133, using CRISPR-Cas9 and western blotting. The *GPR133* gene is associated with glioblastoma, indicating that SNPs within DHSs could be related to brain disorders. These findings suggest that brain-related gene regulatory regions are under adaptive evolution and contribute to the differential expression profiles among primates, providing new insights into the genetic basis of brain phenotypes or disorders between humans and other primates.

## Introduction

Human-specific social and cognitive behaviors, including language, civilization, society, as well as some mental disorders, are rooted in the complex human brain; however, the mechanisms underlying human-specific neurodevelopment remain unclear. Uncovering the link between genetic mutations and human-specific traits and function, by comparison with non-human primates, is a primary goal of brain evolution studies. Previous investigations have provided strong evidence that brain-related genes have important roles in the evolution of brain differences between humans and other primates (Mekel-Bobrov et al., 2005). Differences in primate brains are influenced by many factors, including epigenetic and post-transcriptional modification, and expression profile differences during different stages of brain development (Enard et al., 2002; Cáceres et al., 2003; Brawand et al., 2011).

Unlike coding region variations, which usually lead to loss or gain of gene product function, mutations within non-coding regions can influence the binding affinity of transcription factors or the recruitment of transcriptional elements, thereby affecting the expression of downstream genes. Non-coding regions function in various regulatory processes, including splicing of pre-mRNAs during translation, assisting mRNA localization, and transcription (Le Hir et al., 2003). Xu et al. (2010) analyzed the transcription of intergenic and repeat regions in human, chimpanzee, and macaque brains, and found that intergenic transcripts showed more expression differences among species than exons, indicating the importance of regulatory elements in brain-related differences among species. Several human genomic regions with evidence of accelerated evolution are associated with neurodevelopment, cognition, social behavior, and even brain disorders (Reilly et al., 2015; Doan et al., 2016; Brandler et al., 2018). These findings represent strong evidence of the importance of non-coding regions in primate brain development and divergence, as well as human brain diseases. A more comprehensive analysis should be conducted to determine how brain-related regulatory regions participate in primate brain evolution and what biological functions they serve.

DNase I hypersensitive sites (DHSs) contain a variety of regulatory elements, including promoters, enhancers, silencers, and transcription factor (TF) binding sites (Gross and Garrard, 1988). Moreover, with the development of the ENCODE project (Dunham et al., 2012) and high-throughput DHS detection methods (Crawford et al., 2006; Sabo et al., 2006), hundreds of DHS datasets are available online, providing access to high resolution regulatory network information. In addition, recent studies of DHS evolution (Shibata et al., 2012; Gittelman et al., 2015; Dong et al., 2016; Franke et al., 2017) have proven that these regions have important roles in gene regulation and, therefore, influence human-specific traits. Analysis of the accelerated evolution of regulatory elements controlling brain developmental genes will reveal the genetic mechanisms underlying brain functional differences between humans and non-human primates, providing new insights into human-specific social and cognitive behaviors, as well as identifying associations between genetic mutations and mental disorders.

In this study, DHS data were derived from UCSC (Casper et al., 2018) and another recent study (Shibata et al., 2012). We identified DHSs in the regulatory regions of genes associated with brain development, particularly those brain areas that show more divergence among primates, such as the cortex. Human accelerated DHSs were identified using positive selection analysis methods (Dong et al., 2016), and multiple analyses performed using rapidly evolved DHSs in brain-related gene regulatory regions, to identify rapidly evolving elements which may contribute to the evolution of human biological functions and regulatory mechanisms.

## Results

### Identification of Brain-Related DHSs Under Accelerated Evolution

DHS data from 125 cell types (Table S1) were obtained from UCSC (Casper et al., 2018), along with 15 samples from Duke University, including primary skin fibroblast cells from three human, three chimpanzee, and three macaque individuals, and lymphoblastoid cell lines (B cells immortalized with Epstein-Barr Virus), obtained from the same three human and three chimpanzee individuals, but not the macaques, as EBV does not reliably transfect macaque lymphocytes (Shibata et al., 2012). To focus on regulatory regions correlated with brain development and minimize the influence of constitutive genes in the acceleration analysis, we first filtered out those reported to be housekeeping genes, which are extensively expressed in many tissues and required for basic cellular functions (Eisenberg and Levanon, 2003; Zhu et al., 2008). Next, 243 genes were selected as brain-related genes, using the GO term “brain development” (GO:0007420) (Table S2). The surrounding 50 kb regions, centered on the coordinates of brain-related genes, were defined as their regulatory regions; 184,113 DHSs were discovered in these regions and termed brain-related DHSs.

To identify brain-related DHSs under accelerated evolution, we considered local ancient repeat elements (AREs) to be neutrally evolving (Dong et al., 2016); hence, AREs served as a neutral model for assessing brain-related DHSs with evidence of accelerated evolution (brain-aceDHSs). We obtained the orthologous sequences for each DHS and their corresponding AREs from chimpanzees, gorillas, orangutans, and macaques. After filtering, 39,132 DHSs were suitable for use in the acceleration test, as described our previous report (Dong et al., 2016). The results of acceleration testing indicated that 2,397 DHSs were under accelerated evolution (Table S3). Further, the proportion of brain-aceDHSs (2397/39,132) was significantly higher than that of DHSs across the whole genome, as reported by Gittelman et al. (2015) and our group (Dong et al., 2016; Figure S1, *p* < 2.2e^−16^ and < 2.2e^−16^, respectively; Pearson's Chi-squared test). The enrichment of accelerated DHSs in brain-related related regulatory regions suggests that the regulatory regions of brain-related genes have been under strong positive selection.

**Figure 1:**
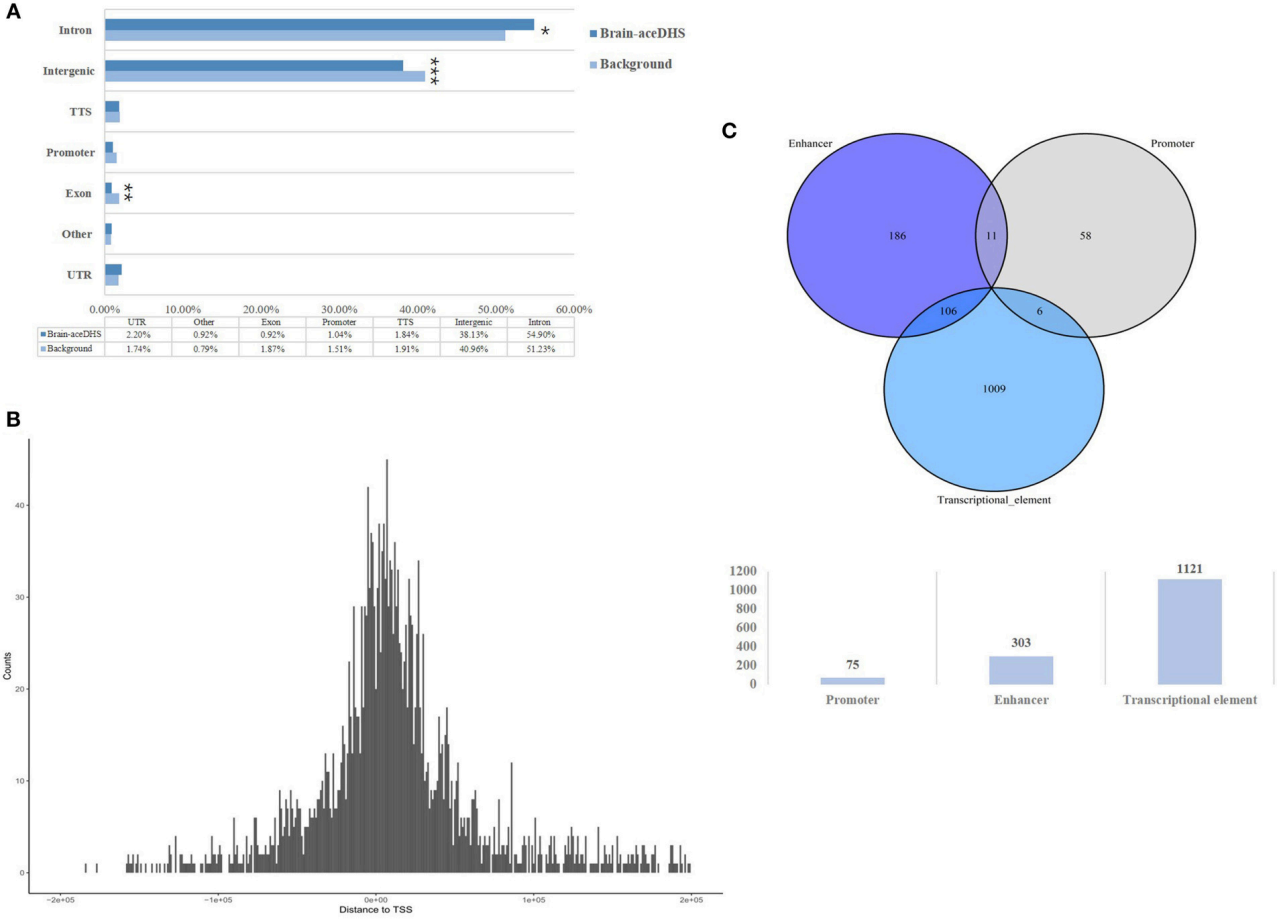
**(A)** Genomic features of brain-aceDHSs. “Other” indicates undefined non-coding region. Brain-aceDHS vs. background: intron, 54.90 vs. 51.23%, *p* = 0.05; intergenic, 38.13 vs. 40.96%, *p* < 2.2e-16; exon, 0.92 vs. 1.87%, *p* = 0.0008; Pearson's Chi-squared test. **(B)** Distance of brain-aceDHSs to TSS. **(C)** Transcriptional features of brain-aceDHSs, according to embryonic stem cell ChromHMM data. ^*^p < 0.05; ^**^p < 0.01; ^***^p < 0.001.

Compared with assessed total DHSs, brain-aceDHSs were significantly enriched in non-coding regions (Figure 1A), consistent with the notion that non-coding regions can evolve and obtain new functions more readily than coding regions. Notably, the contribution of introns to brain-aceDHSs was much higher than that for background DHS (Figure 1A). Introns influence different aspects of gene expression (Le Hir et al., 2003). Moreover, brain-aceDHSs were enriched in regions adjacent to transcription start sites (TSSs), suggesting that brain-aceDHSs are more likely to function as promoters or enhancers (Figure 1B). To investigate this hypothesis, we used embryonic stem cell ChromHMM annotations obtained from UCSC (Casper et al., 2018). We found that 75 brain-aceDHSs overlapped regions designated promoters, 303 overlapped enhancers, and 1,121 brain-aceDHSs overlapped transcriptional progress annotations, including transcriptional transition, elongation, and weakly transcribed (Figure 1C).

These results reveal that the regulatory regions of brain-related genes exhibit significant evidence of accelerated evolution, suggesting that brain-aceDHSs contribute to the adaptive evolution of the human brain. In addition, brain-aceDHSs were enriched for regulatory elements, according to ChromHMM data, suggesting important roles for brain-aceDHSs in the regulation of brain-related genes.

### Brain-aceDHS Target Genes Are Differentially Expressed Among Primates and Enriched in Cell Cycle Process and Cerebral Disorders

To further assess how brain-aceDHSs influence expression of brain-related genes and yield different phenotypes among primates, we identified the target genes potentially regulated by brain-aceDHSs. Chromatin conformation technologies, such as Hi-C (Lieberman-Aiden et al., 2009), can powerfully predict long-range chromatin interactions (Sanyal et al., 2012). Therefore, based on Hi-C data reported by Gittelman et al. (2015), we designated genes that correlated with brain-aceDHSs regions (correlation coefficient >0.7) as targets of brain-aceDHSs. In total, 544 genes (including lncRNAs) were identified as targets of brain-aceDHSs, indicating that these accelerated regulatory regions can control distal genes. Furthermore, in some cases their regulatory profiles indicated that they influenced expression of more than one gene (Table S4).

**Figure 2:**
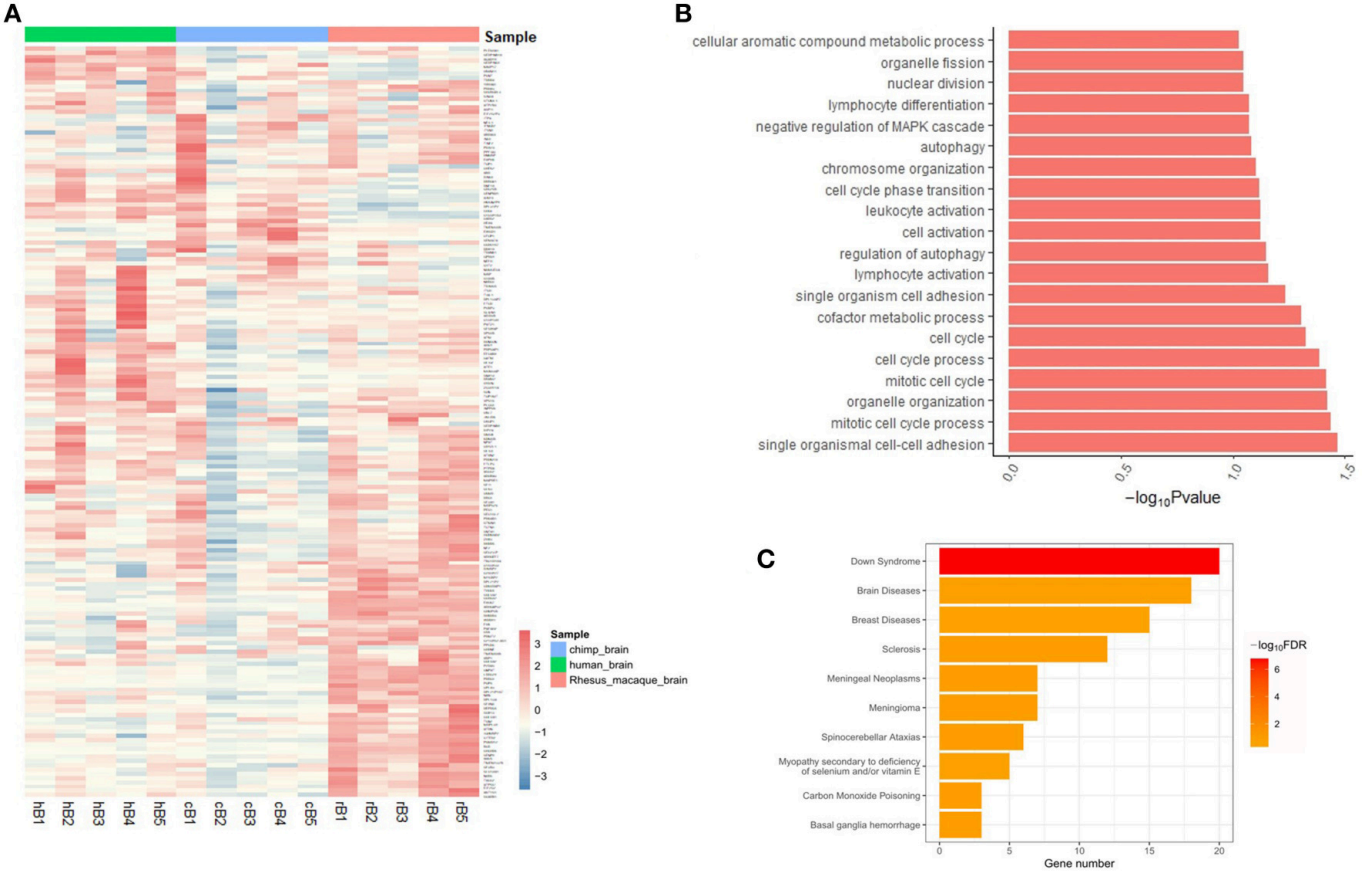
**(A)** Expression profiles of target genes in human, chimp, and macaque brain tissues. Genes with expression data from all three species were selected and normalized Z-scores generated. **(B)** Functional enrichment analysis of brain-aceDHS target genes using DAVID. **(C)** Disease enrichment analysis of brain-aceDHS target genes in the GLAD4U database.

The expression of target genes showed different patterns in brain samples from human, chimp, and rhesus macaque, according to the GSE50782 expression dataset (Figure 2A). Although there were also differences among individuals, it was clear that humans and non-human primates had differential expression patterns. Notably, rhesus macaques exhibited totally different gene expression patterns compared with human and chimp, while some target genes showed similar expression patterns in human and chimp brain samples, indicating the differential expression of these target genes may lead to distinct development progress in different brain areas among primates, which is likely to be the consequence of positive selection on brain-aceDHSs.

Functional annotation analysis showed that the target genes of brain-aceDHSs were mainly enriched for cell cycle process such as mitotic cell cycle process, regulation of autophagy and immune cell activation (Figure 2B). Disease enrichment analysis, based on WebGestalt (Wang et al., 2017), indicated that many of the target genes participate in human diseases (Figure 2C), including brain diseases. Our results indicate that the identified brain-aceDHSs were not only participated in cell proliferation and autophagy which may contributed to traits differences between primates, but also were involved in brain disorders; hence, brain-aceDHSs may harbor causative mutations for these diseases.

### Human-Specific Transcription Factor Binding Sites Within Brain-aceDHSs Are Associated With the Human Brain and Related Diseases

**Figure 3:**
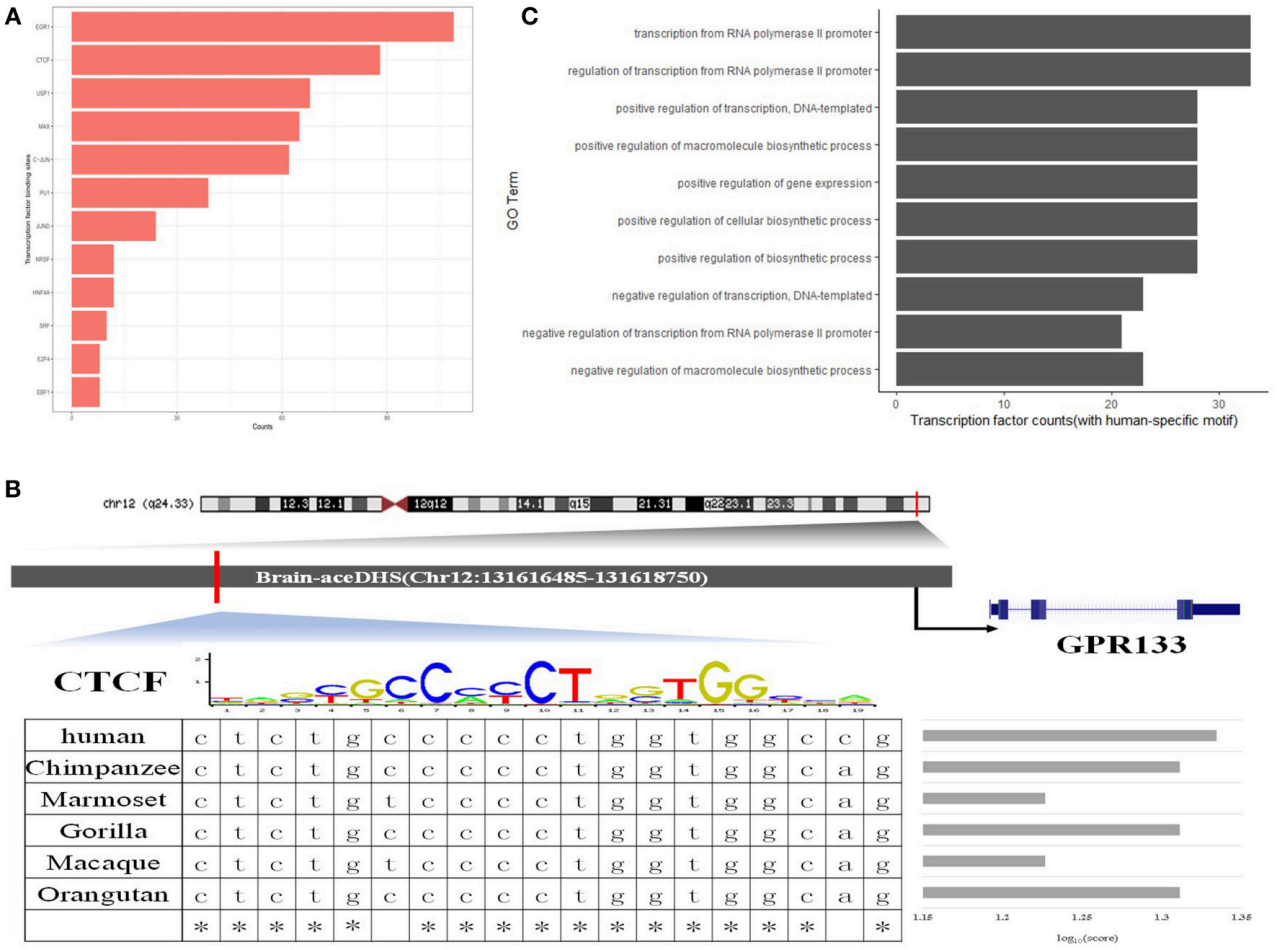
**(A)** Top 10 transcription factor binding sites in brain-aceDHSs, according to the UCSC database. **(B)** Examples of human-specific transcription factor binding sites. **(C)** GO enrichment analysis of human-specific transcription factor binding sites.

To further assess the regulatory functions of brain-aceDHSs, we screened them for TF binding sites using the transcription factor binding site database in UCSC (Casper et al., 2018), We identified 505 TF binding sites in brain-aceDHSs, for factors including EGR1, CTCF, USF1, C-JUN, MAX, and HNF4A, among which EGR1 sites were present at the highest frequency (Figure 3A). EGR1 is an important TF in several biological processes, including cell survival, proliferation, and death. EGR1 expression levels are higher in the mammalian nervous system than those of other inducible TFs (Herdegen and Leah, 1998; Hughes et al., 1999). Moreover, C-JUN is involved in brain development (Mechta-Grigoriou et al., 2003), while CTCF is associated with autistic behavior (Kohler et al., 2017) and intellectual disability (Gregor et al., 2013). These results indicate that brain-aceDHSs contain binding sites for numerous TFs associated with development and the brain, which likely influence brain phenotypes, and brain diseases.

Additionally, we used FIMO to identify potential TF binding sites within brain-aceDHSs in the TF motif database in JASPAR (Mathelier et al., 2016), and selected the potential TF binding motifs with the most significant matches for subsequent analysis. Next, we obtained the corresponding sequences from other primate genomes (chimpanzee, gorilla, orangutan, macaque, and marmoset), based on the homologous coordinates of the identified TF motifs. Sequence comparisons identified TF binding sites with higher or lower binding affinity in the human lineage as human-specific TF binding sites.

One brain-aceDHS (Chr12:131616485–131618750) contained a CTCF binding site and potentially regulates the *GPR133* gene, which is up-regulated in hypoxic conditions and of significance in glioblastoma (Bayin et al., 2016). The binding affinity of CTCF to the brain-aceDHS predicted to regulate *GPR133* was evaluated by JASPAR in different species, and the human branch showed higher affinity compared with non-human primates (Figure 3B). The lower affinity of CTCF for the corresponding regulatory regions within other primate genomes may influence *GPR133* expression. In total, we identified 61 human-specific binding sites within 47 brain-aceDHSs, including for E2F1, PU1, JUND, CTCF, MAX, C-JUN, GABP, C-MYC, SOX17, RREB1, SP1, FOXQ1, E2F6, and USF1 (Table S4). GO enrichment analysis (Figure 3C) indicated that these human-specific TF binding sites play important roles in gene regulation. Some human-specific TFs, such as CTCF, C-JUN, and JUND, also exhibit high expression levels in the brain according to GTEx (Carithers and Moore, 2015), suggesting their importance in the nervous system (Table S4). Moreover, many of these human-specific TFs are also associated with brain diseases (Table S5).

Our results indicate that many TF binding sites within brain-aceDHSs showed different binding affinities across primate lineages, according to the TF binding motif matrix, providing strong support for differences in transcription regulatory functions of brain-aceDHSs that eventually influence expression profiles in the brain, and cerebral disorders, among humans and other primates.

### SNPs in Brain-Related DHSs Provide New Insights Into Potential Brain Disease Causative Variants

Our results (presented above) indicate a notable association between brain disorders (e.g., Alzheimer's disease and schizophrenia) and brain-aceDHSs. Regulatory mutations have previously been reported to underlie human diseases (Weedon et al., 2014). We screened for potential disease-caused SNPs within brain-aceDHSs using GWAS data. GWAS SNP data were downloaded from GWAS Catalog (MacArthur et al., 2017) and GWAS SNPs in brain-DHSs analyzed. We identified 16 GWAS SNPs in 15 brain-aceDHSs that were involved in 19 diseases or phenotypes. Rather than showing preferential associations with brain disorders, brain-aceDHSs were involved in many human diseases, including Tourette syndrome and obsessive-compulsive disorder (Table 1). Among these 16 GWAS SNPs, eight shared the same reported gene, and brain-aceDHS target gene (Table 1). These results are consistent with previous suggestions that SNPs identified by GWAS may contribute to human disease by interfering with the regulatory functions of local DHSs (Maurano et al., 2012). Furthermore, several GWAS SNPs influence traits that are reported to be rapidly evolving, such as type 2 diabetes, body mass index, modified Stumvoll insulin sensitivity index, and obesity-related traits. Mutations within brain-aceDHSs may contribute to these diseases, or rapidly evolved phenotypes, by influencing normal regulatory function.

**Table 1:** GWAS and eQTL SNPs in brain-aceDHSs.

**SNPs harbored in brain-aceDHSs**								
**DHS position**			**SNP Id**	**Target genes**	**Information of GWAS data**		**Information of eQTL data**	
					**Reported gene**	**Traits or diseases**	**Effected gene**	**Tissue**
chr1	112416203	112416371	rs2788612	KCND3-IT1	KCND3	Response to radiotherapy in cancer (late toxicity)	/	/
chr12	112521380	112521596	rs4767364	NAA25	ALDH2	Upper aerodigestive tract cancers	/	/
chr12	121426887	121427060	rs12427353	HNF1A	HNF1A	Type 2 diabetes	/	/
chr12	124499346	124500728	rs1048497	ZNF664	ZNF664	Visceral adipose tissue adjusted for BMI, Visceral adipose tissue/subcutaneous adipose tissue ratio	ZNF664	Heart
chr12	124800542	124801674	rs1809889	MIR6880	FAM101A	Height	/	/
chr12	131621602	131623539	rs885389	RAN, GPR133	GPR133	RR interval (heart rate)	/	/
chr13	77552178	77552448	rs11149058	CLN5	MYCBP2, KCTD12, FBXL3, CLN5, BTF3P11, IRG1	Tourette's syndrome or obsessive-compulsive disorder	/	/
chr13	110755284	110755930	rs16854	–	COL4A1, COL4A2	Night sleep phenotypes	/	/
chr17	4667920	4668143	rs193042029	TM4SF5	TM4SF5	Triglycerides	/	/
chr18	60845823	60846206	rs12454712	BCL2	BCL2	Body mass index, Waist-to-hip ratio adjusted for body mass index, Modified Stumvoll Insulin Sensitivity Index	/	/
chr18	61145663	61146021	rs79285331	SERPINB5	NR	PR interval in Tripanosoma cruzi seropositivity	/	/
chr21	47690016	47690212	rs2839186	MCM3AP	MCM3AP	Testicular germ cell tumor	MCM3AP	Testis
chr15	75339313	75339899	rs78664321	PPCDC	/	/	PPCDC	Brain
chr17	531671	532233	rs331014	VPS53	/	/	VPS53	Brain
chr20	3733637	3734366	rs45495794	C20orf27	/	/	C20orf27	Brain
chr22	30218202	30218476	rs73394831	ASCC2	/	/	ASCC2	Brain
			rs17711461	ASCC2	/	/	ASCC2	Brain

**Figure 4:**
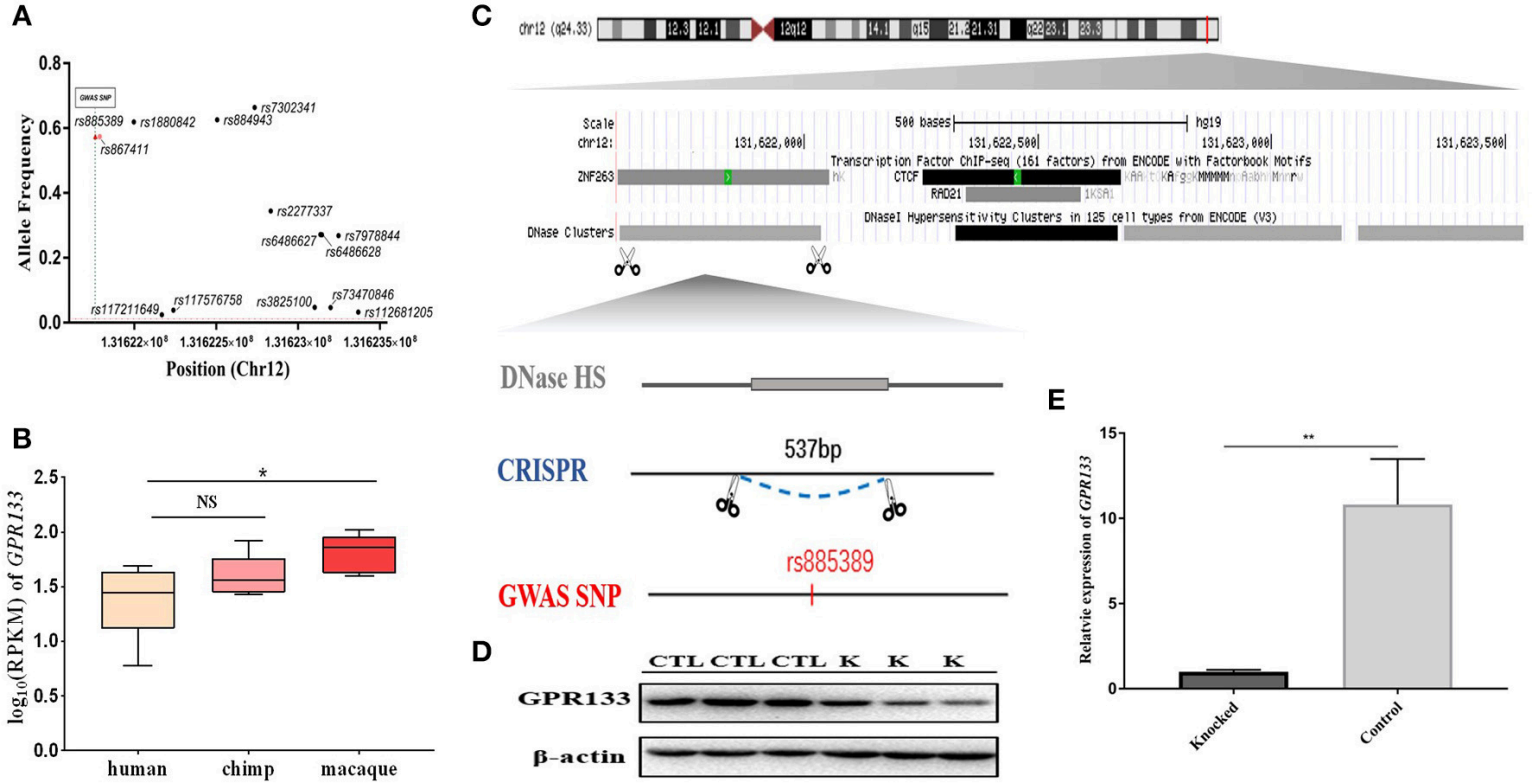
Knockout of a brain-aceDHS that potentially regulates GPR133. **(A)** Data from the 1,000 Genomes Project demonstrate that there are SNPs with allele frequencies >1% within the knocked out brain-aceDHS. **(B)** Expression of *GPR133* in human, chimp, and macaque brain tissue. **(C)** The position of the knocked out aceDHS in the genome. **(D)** Results of GPR133 western blotting experiment before and after brain-aceDHS knock out. “K,” DHS-knockout cells; “CTL,” control 293T cells. **(E)** The expression of GPR133 before and after aceDHS knockout. ^*^p < 0.05; ^**^ *p* < 0.01; ^***^p < 0.001.

One brain-aceDHS (chr12:131621602–131623539) contained the GWAS-identified SNP rs885389, and according to 1,000 Genomes Project data, 14 SNPs with >1% allele frequency are present in this brain-aceDHS, including rs885389 (Figure 4A, Table S6). Within these SNPs, there was high LD (linkage disequilibrium) between rs885389 and rs867411 (Table S6), indicating that it is not fixed in the population and likely to become a population-specific disease target.

In addition, we found that expression of the target gene, *GPR133*, differed among primates, according to expression profiles in the GSE50782 dataset, with significant differential expression between human and macaque (Figure 4B; *p* = 0.0489, *T*-test), supporting function of this brain-aceDHS in regulation of *GPR133*. Using the CRISPR/Cas9 system, we successfully knocked out part of this brain-aceDHS in the 293T cell line, which was identified by sequencing (Figure 4C, Figure S2), and found that this knockout resulted in significant down-regulation of *GPR133* expression (Figure 4E; *p* = 0.0082, *T*-test). Further, western blot assays showed that *GPR133* protein expression was decreased in knockout cells (Figure 4D), suggesting that this brain-aceDHS may serve as a *GPR133* enhancer, and that variations within it may influence *GPR133* expression during pathogenesis.

Furthermore, we used eQTL data from UCSC (Casper et al., 2018) to further explore the impact of mutations within brain-aceDHSs. The results showed that 82 SNPs were contained in brain-aceDHSs that influenced gene expression in brain tissues (Table S7). Among them, target genes of five SNPs (rs78664321, rs331014, rs45495794, rs73394831, and rs17711461) were same as the reported genes from eQTL database and exhibited differential expression between primates according to expression profile of GSE50782 dataset, confirming the regulatory functions of these motifs (Table 1, Figure S3, *T*-test). Notably, the mutated allele of one eQTL SNPs rs17821294 was the same as in chimp (T>G/T, chimp allele displayed first, then “>,” then human alleles). The mutated allele frequency of rs17821294 was 7.9%, which tend to differ from that of chimp in human population. And expression data from GSE50782 dataset showed that affected gene of rs17821294, INPP5K, significantly differential expressed (*p* = 0.034, *T*-test) between human and chimp, but not in macaque which allele genotype was C (Figure S4), indicating that these eQTL SNPs in brain-aceDHSs may play key roles in the differential expression of brain-related genes.

## Discussion

In this study we performed a comprehensive analysis of the regulatory regions of brain-related genes to assess how these impact human brain evolution. We examined the evolutionary characteristics of brain development-related DHSs among primates. Of 39,132 identified DHSs, 2,397 had undergone accelerated evolution and were considered brain-aceDHSs. The higher brain-aceDHS ratio in the regulatory regions of brain-associated genes, relative to the accelerated DHS ratio across the whole genome (Gittelman et al., 2015; Dong et al., 2016), indicates that, in addition to the rapid evolution of nervous system genes (Dorus et al., 2004), the regulatory regions of brain-associated genes are also evolving rapidly in primates. The genomic features of brain-aceDHSs confirmed their importance in transcription progress and their consequent influence on human-specific traits through regulation of target genes. The brain-aceDHSs identified here may help to reveal how humans have acquired specialized cerebral functions during evolution.

We identified target genes of each brain-aceDHS using Hi-C, which can locate long-range contacts between two genomic areas. Target genes were enriched for the biological processes, regulation of endopeptidase activity, and brain development, indicating their biological functions are related to brain traits. According to expression profiles in primate brain tissues from GEO data, target genes showed differential expression patterns among human, chimp, and macaque, suggesting that expression of target genes regulated by brain-aceDHSs eventually leads to the remarkable differences between humans and other primates during brain-associated development. Notably, the differences in target gene expression profiles between human and chimp were less marked than those between human and macaque, consistent with the fact that the macaque brain has diverged further from those of human and chimp. Our results provide evidence that the differential expression of target genes may contribute to primate brain divergence and that brain-aceDHSs could be the main driver of this phenomenon. And functional analysis showed that target genes were mainly enriched for cell cycle process such as mitotic cell cycle process, regulation of autophagy, and immune cell activation. Considering target genes were also mainly expressed in brain, we assumed that these brain-aceDHSs were mainly participated in cell proliferation and programmed death during brain development or neural cell renewal.

In addition, positive selection acting on brain-aceDHSs is mainly reflected in differences in TF binding affinity among primates. We identified 61 recognized TF binding sites, with potentially human-specific binding affinity, that are enriched in important transcriptional events, providing strong evidence for the impact of brain-aceDHSs on transcription of differentially expressed target genes. Moreover, the majority of identified TFs were associated with brain-related diseases and brain dysfunction, such as CTCF (Gregor et al., 2013), JUND (Herdegen and Leah, 1998), BPTF (Mu et al., 1997), NRSF (Schoenherr and Anderson, 1995; Schiffer et al., 2014), and SP1 (Citron et al., 2008). For example, TFAP2B is a member of the AP-2 family of TFs and is associated with neurological pathways and various mental disorders (Mani et al., 2005; Nilsson et al., 2014). Further, the human-specific TF binding site for TCF4 is involved in the initiation of neuronal differentiation; according to the Uniprot database, TCF4 is highly expressed in the brain. In addition to its biological function in brain development, TCF4 is also associated with speech and language abilities, as well as intellectual disability (Maduro et al., 2016). These data indicate that, through these human-specific TF binding sites, brain-aceDHSs impact brain regulatory networks involved in brain-related, and even maintenance, functions in humans and other primates, eventually leading to phenotypical differences among species. The biological functions of the identified human-specific TF binding sites included participation in brain disorders, providing new insights into the pathogeneses of species-specific brain disease.

Regulatory regions harbor many disease-causing mutations. Subsequently, we analyzed brain-related DHSs in the GWAS database, to investigate the association between brain-aceDHSs and human diseases. GWAS SNP rs11149058 is associated with Tourette syndrome and obsessive-compulsive disorder, and the target gene of the corresponding DHS containing this SNP, *CLN5*, was included among genes reported in GWAS analyses. Our results suggest that this SNP may affect the expression of *CLN5*, which is involved in disease pathogenesis. Rapid evolution of brain-related DHSs can further illustrate the relationship between humans and non-human primates, in terms of brain evolution as a consequence of genotype variations that result in phenotypic differences. Several GWAS SNPs associated with obesity were observed among brain-aceDHSs, suggesting that obesity-related traits in humans may have undergone rapid evolution.

In addition to GWAS SNPs, we identified 82 eQTL SNPs that overlapped with brain-aceDHSs in brain tissue. Many affected genes participated in brain-related diseases or disorders; for example, inactivation of *PNKP* causes brain dysplasias, such as microcephaly and cerebellar atrophy (Bras et al., 2015), while *VPS53* is associated with cerebral and cerebellar atrophy (Feinstein et al., 2014; Kohler et al., 2017). Further, *PLD2* is not only involved in neurotransmission and neurodevelopment, but also affected in neurological disease (Gratacòs et al., 2009; Ghim et al., 2016), while *ATF5* is an important regulator of cerebral cortex formation, which functions in cerebral cortical neuroprogenitor cells to maintain their proliferation and block their differentiation into neurons; *ATF5* is also associated with bipolar disorder (Kakiuchi et al., 2007). The associations of genes influenced by brain-aceDHS SNPs with brain disorders strongly suggests that these elements regulate target genes, causing differences in traits and even resulting in brain disorders. The SNPs, rs5759617, rs713682, and rs737819, which have minor alleles in humans the same as the alleles found in non-human primates, affected expression of the GWAS reported gene, *RAB36*, indicating that the risk allele frequency varied among different populations, causing different pathogeneses, and conferring different levels of risk for their corresponding diseases.

The current study has limitations. We may have missed some target genes of brain-aceDHSs subject to remote regulation. Besides, limited to the current expression profiles of primate brain tissue, still many target genes were undetectable, and specific verification is needed. Further, confirmatory experiments are needed to validate our findings, and we are planning to undertake such research using expanded human stem cells. Our research suggested that brain-related gene regulatory regions are under adaptive evolutionary pressure, contributing to their differential expression profiles among primates, and providing new insights into the genetic basis of disease or brain-associated variation in regulatory regions of brain-related genes, between humans and other primates.

## Materials and Methods

### Definition of Brain-Related Genes and Their Corresponding Regulatory Regions

Candidate genes were obtained from AmiGO2 using the filter parameters “forebrain development” or “cerebral cortex development,” and choosing “*Homo sapiens*” as the species of interest. The reason for selecting these two parameters was that the forebrain and cerebral cortex have clear morphological differences (Finlay et al., 2001), indicating that they have diverse functions in primates. Then, we combined the genes identified using the two parameters and filtered out those reported to be housekeeping genes, which were consistently expressed in numerous tissues or essential to basic cellular function, with the aim of focusing on brain-specific genes. After filtering, 243 genes remained, and we designated them brain-related (Table S1). Using NCBI, we obtained the coordinates of brain-related genes in the human genome sequence (hg19 version). The regulatory regions of each brain-related gene were delineated as sequences within ±50 kb from the center of the gene.

### Identification of Brain-Related DHSs and Data Pre-processing

Total DHS data (track: wgEncodeRegDnaseClusteredV3) were obtained from UCSC, along with data reported from Duke University, including from primary skin fibroblast cells from three human, three chimpanzee, and three macaque individuals and lymphoblastoid cell lines from the same three human and chimpanzee individuals, but not the macaques, as EBV does not reliably transfect macaque lymphocytes (Shibata et al., 2012). All these data were converted to hg19 using the UCSC liftover tool, for consistency. Single base-pair DHS data were removed as they were not recognized in subsequent processing. DHS data from different cell types were merged using BEDOPS (Neph et al., 2012) with the command “bedops –merge.” Then, we intersected the processed DHS data and regulatory regions of brain-related genes using BEDOPS, with the command “bedops -element-of 1.” A total of 184,113 DHSs located in the regulatory regions of brain-related genes were identified and termed brain-related DHSs.

For each brain-related DHS, we obtained ortholog DHS sequences and local ancient repeat elements (AREs) within ±5 kb from the center of the corresponding DHS in the gorilla, chimpanzee, orangutan, baboon, and macaque genomes. First, sequences <100 bp were filtered out, leaving 155,921 DHSs, 16,202 LINE1, and 7,993 LINE2 sequences. The following were also filtered out: DHSs and AREs with ortholog sequences in <4 species; DHSs where the background ARE was within 5 kb in human and >5 kb away from the center of the DHSs in the genome of any other primates used; where the length of DHSs was greater than that of background AREs, or the DHS overlapped with its ARE in any genome; and DHSs whose local AREs contained potentially non-neutral LINEs (i.e., that overlapped with coding exons, promoters, simple repeats, low complexity regions, or segmental duplications). Finally, 39,132 DHSs were selected for subsequent analysis.

### Acceleration Analysis of Brain-Related DHSs

Multiple sequence alignments (MSAs) of DHSs and AREs were constructed using Muscle (Edgar, 2004). Phylogenetic trees were constructed from MSAs using phyloFit (Siepel and Haussler, 2004) and phyloP was used to assess whether DHSs within a human sub-branch were under accelerated evolution, by assuming that AREs were under neutral evolution. Specifically, the SPH model was applied in phyloP, and both the “sub-branch” and the “sub-branch given the whole tree” tests conducted. If the *P*-values for both tests were significant (FDR-adjusted *p* < 0.05), then the DHS was considered an aceDHS. In cases where human AREs were more than twice the length of human DHSs, a sliding window was applied to generate multiple sub-AREs from the original ARE by setting the window and step length to be the DHS length and 10% of the DHS length, respectively. Then, the aforementioned procedures were applied to compare the DHS with each of the sub-AREs, and a DHS was considered an ace-DHS if it was significant in more than half of cases.

### Annotation of Brain-aceDHSs

Annotations generated by the analysis of brain-aceDHSs were added to the human gene annotation file (hg19) and analyzed in Homer using annotatePeaks.pl, with default parameters. The distance of each brain-aceDHS from the TSS was calculated from the center of the DHS to the nearest TSS. If the assigned TSS was located upstream, the distance was a negative number.

Embryonic stem cell ChromHMM annotations were obtained from UCSC (track: wgEncodeBroadHmmH1hescHMM) and marks overlapping with brain-aceDHSs selected.

### Brain-aceDHSs Target Gene Enrichment Analysis

Target genes that could be uniquely mapped to Ensembl ID were selected. Then, we carried out functional enrichment analysis for the remaining target genes via the DAVID website (https://david.ncifcrf.gov/), with the target genes of unselected DHSs as background (Table S4). The most significant GO terms from the molecular function, biological process, and cellular component categories were selected to generate a condensed list of enriched GO terms.

Disease enrichment analysis was conducted with WebGestalt (Wang et al., 2017) using the GLAD4U database and the over-representation analysis enrichment method. All mapped Entrez Gene IDs from the human genome were used as background. Disease terms with the highest number of target genes were selected to produce a condensed list of enriched diseases.

### Expression Profiles of Target Genes in Primate Brain Tissue

Expression profiles were obtained from GEO, using the keywords “brain” and “primates.” The GSE50782 dataset of expression profiles was selected and only data from human, chimp, and macaque brain tissue chosen for subsequent analysis. Target genes with expression data for all three species were selected and normalized using Z scores. The pheatmap package in R was used to cluster differentially expressed genes.

To identify differences in expression of *GPR133* among primates, *GPR133* expression profiles were selected from the GSE50782 dataset and plotted using boxplot. *T*-tests were conducted to evaluate the significance of differences among expression levels in human vs. chimp and human vs. macaque (*p* = 0.294 and 0.0489, respectively).

### Analysis of Regulatory Features in Brain-aceDHSs

Regulatory features and transcription binding sites within brain-aceDHSs were obtained from UCSC (track: wgEncodeRegTfbsClustered). Various transcription binding site footprints overlapping with DHSs were selected.

Potential TF binding sites were scanned using FIMO with default parameters, an output threshold of 0.0001, and transcription binding position weight matrix data from JASPAR (JASPAR2018_CORE_vertebrates_non-redundant.meme). To identify human-specific TF binding sites, we downloaded EPO alignments for the six primates based on TF binding site coordinates from Ensembl. Using motifs in the JASPAR database, we assessed whether the obtained TF binding sites had higher or lower affinity in the human lineage compared with non-human primates. Binding affinity was evaluated according to scores generated using the JASPAR scan program, with an 80% relative profile score threshold. GO enrichment analysis of human-specific TF were conducted by WebGestalt using human whole genome as background and ORA (over-representation analysis) enrichment method.

### SNPs Contained in Brain-Related DHSs

GWAS SNP data were downloaded from GWAS Catalog and SNP coordinates transformed into hg19 using LiftOver software, for consistency with brain-aceDHSs. Next, SNPs within brain-related DHSs were identified. Finally, we compared reported genes and target genes of corresponding DHSs to assess whether GWAS SNPs contribute to human diseases or phenotypes by affecting the regulatory functions of DHSs.

In addition, eQTL data (filename: gtexEqtlCluster) were downloaded from UCSC, version hg19. Then, all SNPs within brain-aceDHSs, as well as those expressed in brain tissue, were selected.

## Brain-aceDHS Knockout Experiments

We designed sgRNAs based on the sequence of the brain-aceDHS (chr12:131621602–131623539) to knock out this regulatory region in the 293T cell line. The vector, pCMV-Cas9, which has a selectable neomycin marker, was obtained from Addgene (41815).

sgRNAs were designed via the Zhang Lab website (https://zlab.bio/guide-design-resources), as follows: sgRNA1, 5′-CCTTTCCGAAAGGTCACAGGAGC-3′, and sgRNA2, 5′-CCTGCCCGGTCCATCTCAGTGGC-3′. These sgRNAs were then cloned into the corresponding U6-sgRNA plasmid vectors. A total of 1 μg of DNA (0.25 μg sgRNA1 vector + 0.25 μg sgRNA2 vector + 0.5 μg Cas9-vector) was diluted in 50 μl DMEM medium and 3 μl Genjet reagent (GenJet™, SL100489) was also diluted in 50 μl DMEM medium, then the two solutions were mixed together and incubated for 20 min. sgRNA and Cas9-vectors (0.25 μg sgRNA1 vector + 0.25 μg sgRNA2 vector + 0.5 μg Cas9-vector) were co-transfected into the 293T cell line using Genjet (GenJet™, SL100489). After 48 h, 600 μg/ml G418 was added to the medium and replaced every 48 h. Single colonies were selected using the limiting dilution method 8 days after addition of G418 and verified by PCR and sequencing.

### qPCR

The expression of *GRP133* was detected by qPCR using the following primer pair: 5′-AGGAAAAGGGAGTCACGCTTC-3′ and 5′-GTCATGGAATTGTCCCGCGTA-3′. β-actin was used as a reference gene for qPCR analysis. The reagents for the qPCR experiment were from TAKARA (DRR096A). The relative expression data of GRP133 from wild type and knock-out samples (knock-out sample had six replicates, wild type had eight replicates) was calculated using formula: relative expression = 2^−ΔCt^ and was normalized based on knock-out samples. *P*-values calculated by *T*-test (*p* = 0.0082).

### Western Blotting

Cells were lysed in 300 μl of radioimmunoprecipitation assay buffer (Biotech well; Shanghai, China) containing 1 mM PMSF per well. Total protein concentrations were determined by bicinchoninic acid assay (Biotech well; Shanghai, China) and samples normalized with lysis buffer, then mixed with an equal volume of 2 × Laemmli sample buffer, and solubilized by boiling for 10 min at 99°C. Proteins were separated by SDS/PAGE and tagged proteins detected using mouse monoclonal antibody against GPR133 at the dilution recommended by the manufacturer. β-actin was detected using mouse monoclonal anti-beta actin antibody (EarthOx; San Francisco, CA).

## Data Availability

Publicly available datasets were analyzed in this study. This data can be found here: “http://genome.ucsc.edu/index.html”.

## Author Contributions

FL, WT, and CC: research design and supervision; YrL, XW, WW, YO, and CH: data analysis; HY, ZJ, JL, BC, YiL, and LH: experiments; YrL and FL: writing.

### Conflict of Interest Statement

The authors declare that the research was conducted in the absence of any commercial or financial relationships that could be construed as a potential conflict of interest.

## Supplementary Material

Figure S1 Proportions of DHSs under accelerated evolution contributions (DHSs under accelerated evolution vs. total DHSs selected for analysis).

Figure S2 Clone of selected DHS-knocking 293T cells and the sequencing result.

Figure S3 Expression of target genes of five SNPs (rs78664321, rs331014, rs45495794, rs73394831, and rs17711461).

Figure S4 Information of eQTL SNPs rs197440 and rs17821294 and the expression of the corresponding affected genes.

Table S1 List of 125 cell type sources from UCSC.

Table S2 List of identified brain-related genes.

Table S3 DHSs used for acceleration analysis and the identified aceDHSs.

Table S4 Ace-brain DHS target genes.

Table S5 Potential human-specific transcription factor binding sites in brain-aceDHSs.

Table S6 Information of 14 SNP obtained from 1,000 Genome Project and pairwise LD calculation result of rs885389 and rs867411 using Ensembl website.

Table S7 SNPs within brain-aceDHSs, according to eQTL data from UCSC.
