# Supplementary figures and images for: Evolution and Comprehensive Analysis of DNaseI Hypersensitive Sites in Regulatory Regions of Primate Brain-Related Genes
Source: Front Genet. 2019 Mar 7;10:152. doi: 10.3389/fgene.2019.00152 (PMC6423895; doi:10.3389/fgene.2019.00152)

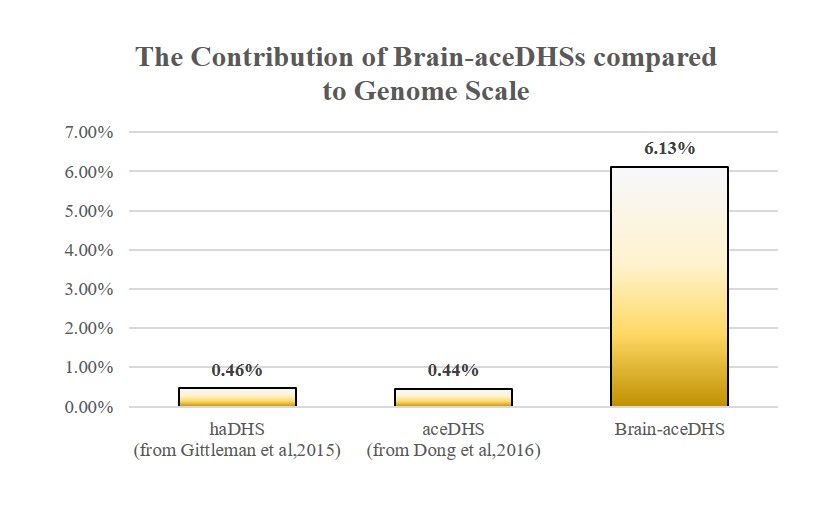

Supplement: Figure S1 — Proportions of DHSs under accelerated evolution contributions (DHSs under accelerated evolution vs. total DHSs selected for analysis). [file Image_1.JPEG]

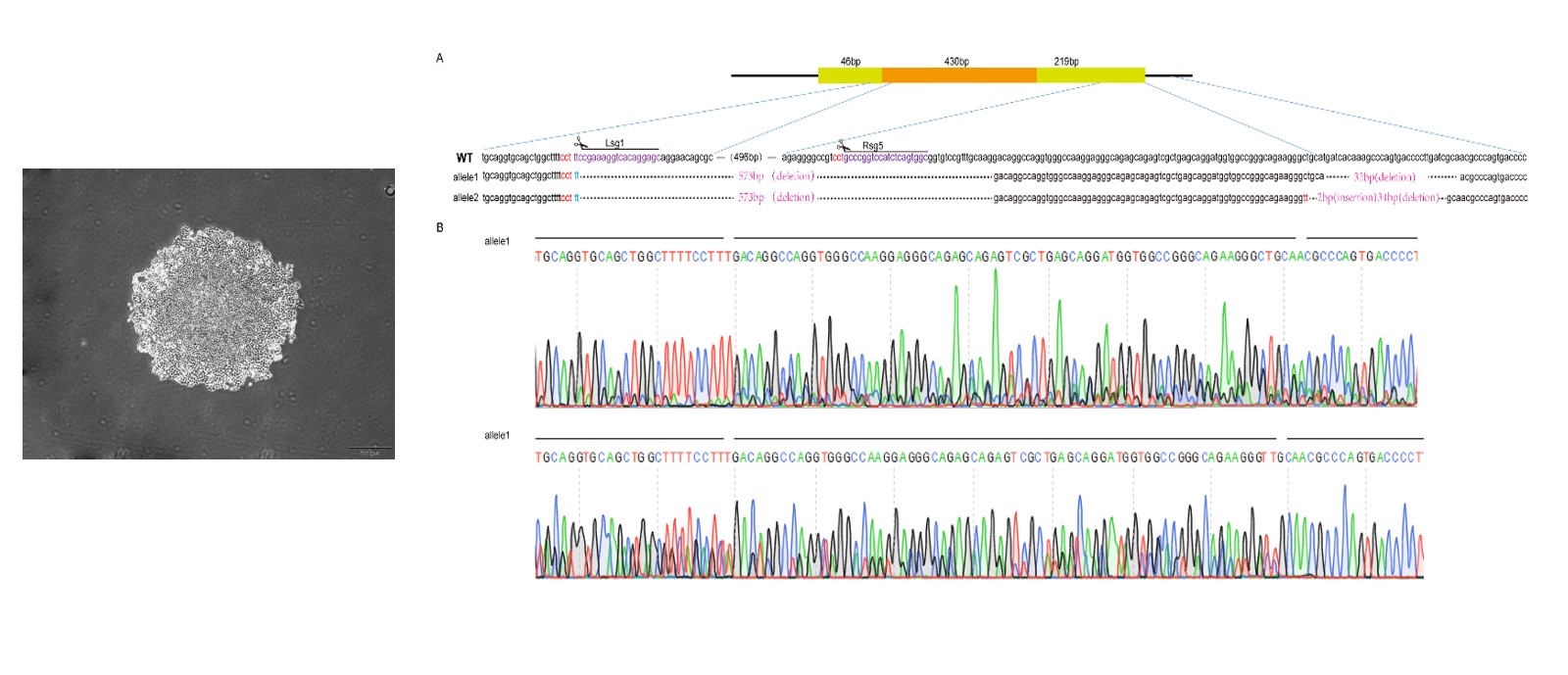

Supplement: Figure S2 — Clone of selected DHS-knocking 293T cells and the sequencing result. [file Image_2.JPEG]

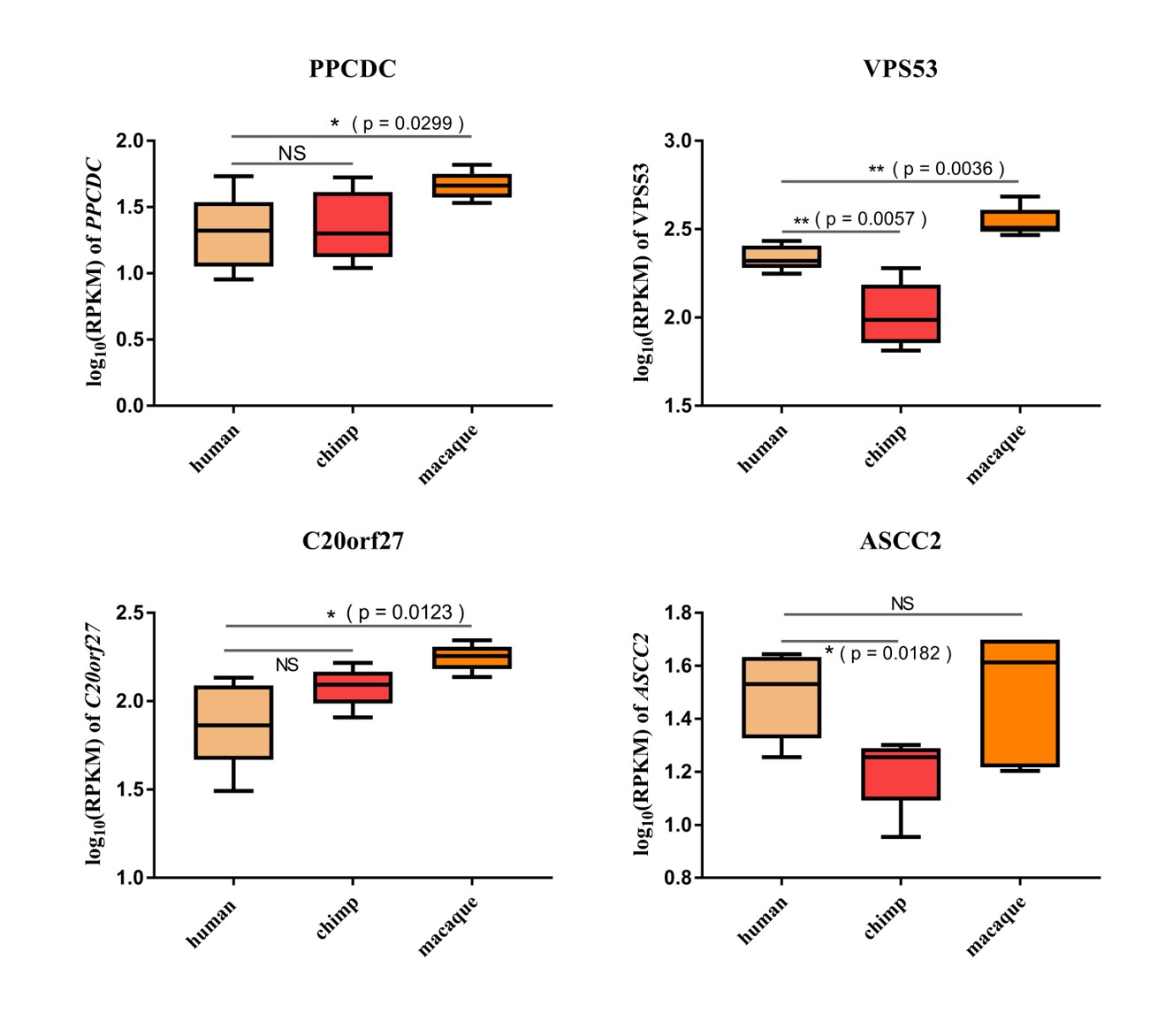

Supplement: Figure S3 — Expression of target genes of five SNPs (rs78664321, rs331014, rs45495794, rs73394831, and rs17711461). [file Image_3.JPEG]

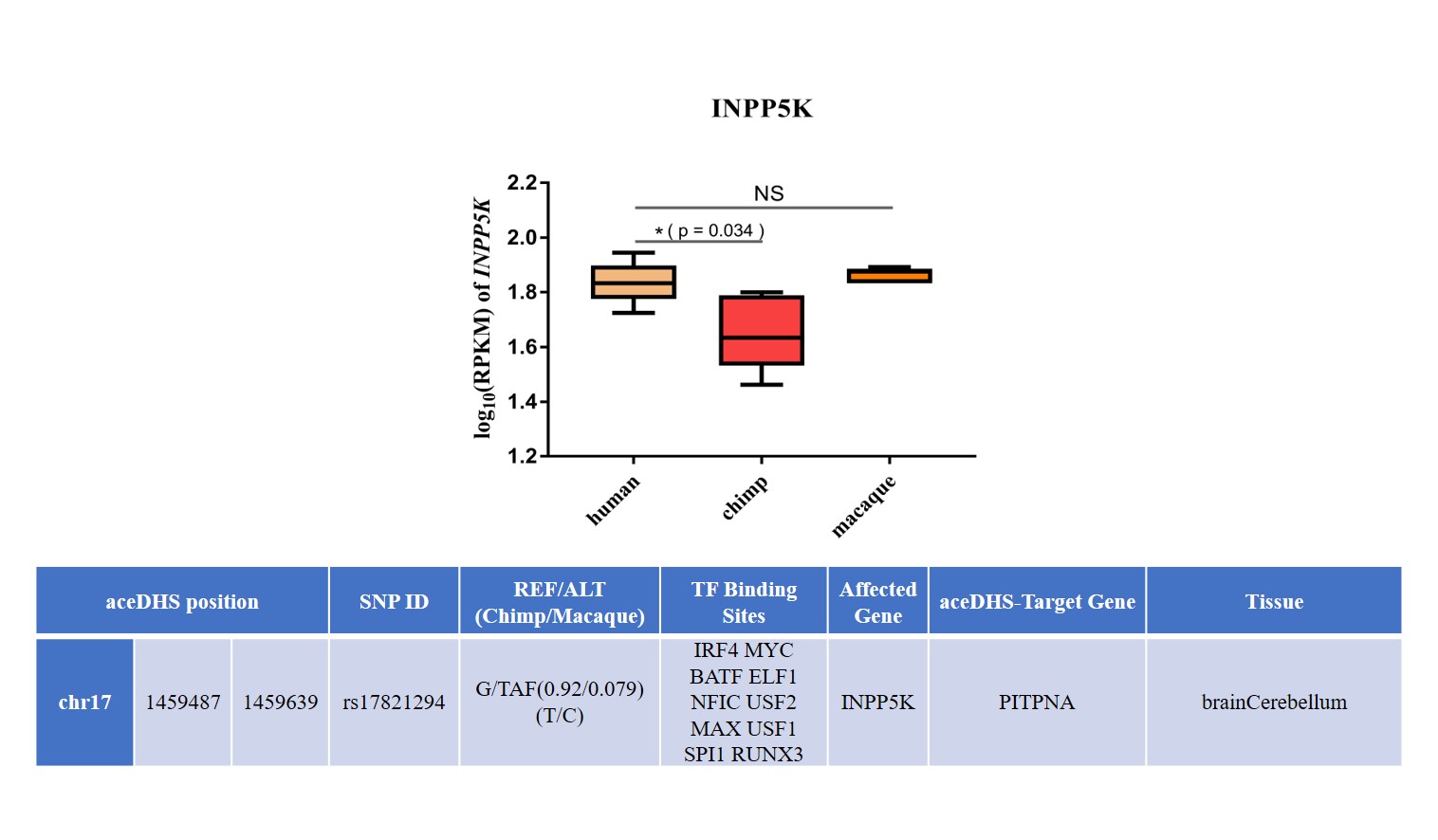

Supplement: Figure S4 — Information of eQTL SNPs rs197440 and rs17821294 and the expression of the corresponding affected genes. [file Image_4.JPEG]
